# GmPP2C113, a Soybean Protein Phosphatase, Positively Regulates Both Salt Tolerance and Symbiotic Nodulation

**DOI:** 10.3390/genes17070815

**Published:** 2026-07-17

**Authors:** Danxia Ke, Zhaoyuan Zhou, Xiaoli Song, Jianuo Lin, Kexin Zhang

**Affiliations:** College of Life Sciences, Xinyang Normal University, Xinyang 464000, China; zzy_0203@xynu.edu.cn (Z.Z.);

**Keywords:** soybean, protein phosphatase PP2C, protein interaction, salt stress, nodulation

## Abstract

Background/Objectives: Protein phosphatase type 2C (PP2C) family members are key signaling hubs in plants, but their roles in mediating the trade-off between stress adaptation and symbiotic interactions remain unclear. This study aimed to investigate the function of soybean GmPP2C113, which was identified as a potential regulator linking salt stress responses and symbiotic nodulation, in coordinating these two biological processes in soybean (*Glycine max*). Methods/Results: The *GmPP2C113* gene was cloned, and its subcellular localization, transcriptional activity, and protein interactions were characterized. Expression patterns under salt stress and rhizobial inoculation were analyzed. The biological role of *GmPP2C113* was assessed using transgenic soybean plants overexpressing *GmPP2C113*. GmPP2C113 was localized in the nucleus and possessed transcriptional activation capability and interacted with GmPP2C47. Its expression was strongly induced by salt stress and by rhizobial infection, with the highest levels detected in mature nodules. Overexpression of *GmPP2C113* significantly enhanced salt tolerance, upregulated stress-related genes, increased nodule numbers, and promoted symbiotic nodulation under salt stress by inducing nodulation marker genes. Conclusions: These results identify *GmPP2C113* as a positive modulator of salt tolerance and reveal its novel role as a molecular node that sustains symbiotic nodulation under salt stress. This provides insight into the coordinated regulation of stress adaptation and symbiosis in soybean.

## 1. Introduction

Abiotic stresses such as drought, temperature extremes, and heavy metal pollution disrupt plant growth through multiple pathways. Drought stress inhibits photosynthesis and disrupts osmotic regulation [1]. High-temperature stress impairs photosynthetic efficiency and membrane integrity [2]. Low-temperature stress damages the photosynthetic apparatus [3], while heavy metal stress induces ROS accumulation and programmed cell death [4]. In particular, salt stress not only induces osmotic stress that hinders root water uptake, but also causes ionic imbalance and oxidative stress, leading to metabolic disorders and even plant death [5]. With climate change intensifying, abiotic stresses increasingly limit crop yield and quality, among which salt stress poses a major threat to crop production worldwide.

The nitrogen-fixing symbiosis between legumes and rhizobia is a core component of the nitrogen cycle in agricultural ecosystems, possessing irreplaceable production and ecological value. As a globally important legume crop, soybean converts atmospheric nitrogen into ammonia through root nodules, providing high-quality protein resources for humans and livestock while effectively increasing soil nitrogen reserves and significantly reducing the demand for chemical nitrogen fertilizers [6,7]. However, this finely regulated symbiotic process is highly sensitive to environmental perturbations. Salt stress, as a major limiting factor, interferes with multiple stages of nodulation, from rhizobial infection and nodule primordium formation to nitrogen fixation maintenance [8,9,10,11,12], ultimately leading to reduced nodule numbers and diminished nitrogen fixation efficiency [13,14,15].

The inhibitory effects of salt stress on soybean nodulation have been documented for over five decades [16], with soybean nodules being particularly vulnerable during early developmental stages, as evidenced by marked reductions in nodule weight, number, and dry matter accumulation [17]. Mechanistically, salt stress impairs nodulation from both host and rhizobial perspectives. On the host side, salt stress reduces root daidzein secretion, which in turn represses rhizobial nodC expression and delays primary-root nodulation [18]. On the symbiotic side, salt stress hinders oxygen uptake by bacteroids, weakens bacteroid respiration, and inhibits nitrogen fixation activity [19], while also triggering the degradation of leghemoglobin in nodules and thereby compromising nitrogenase function [20]. Despite these physiological and phenotypic observations accumulated over decades, the underlying genetic and molecular mechanisms by which salt stress suppresses symbiotic nodulation remain largely unknown and await systematic elucidation.

Recent studies have greatly advanced our understanding of the molecular mechanisms involved. Studies have found that GSK2-8, a member of the glycogen synthase kinase 3 family, can directly phosphorylate the transcription factor GmNSP1a, inhibiting its transcriptional activity, thereby mediating the negative regulation of soybean nodulation in saline environments [21]. Concurrently, the NAC transcription factor *GmNAC181*, which is strongly induced by salt stress, promotes nodulation by binding to the *GmNIN1a* promoter and stimulating its expression, playing a positive regulatory role [22]. Furthermore, the ABA signaling pathway is deeply involved in the interactive network regulating salt stress and nodulation [23]. ABA inhibits bacterial infection and the transcription of nodulin genes [24,25]. Overexpression of the core ABA signaling component *ABI5* enhances stress sensitivity, while the dominant-negative allele mutant abi1 of *ABI1* exhibits ABA insensitivity and a super-nodulation phenotype; deletion of *ABL1* also attenuates ABA responses [26,27,28]. These discoveries reveal a molecular pathway regulating nodulation under salt stress, composed of GSK3 kinase, NAC transcription factors, and ABA signaling components. However, the complete regulatory network remains to be further elucidated. In-depth investigation of the molecular mechanisms by which salt stress inhibits soybean nodulation will provide theoretical support for breeding new soybean germplasms with high salt tolerance and efficient nitrogen fixation, which is of great significance for promoting sustainable green agricultural development.

Protein Phosphatase 2C (PP2C) is an important class of protein phosphatases widely present in plants, animals, and microorganisms, functioning in various biological pathways including cellular signaling, growth and development, and stress adaptation [29,30,31]. From an evolutionary perspective, the number of genes in this family increases significantly as plants evolve from lower to higher forms: green algae possess only about 10, mosses and lycophytes have nearly 50, while in higher plants, the family has expanded substantially, with Arabidopsis, rice, maize, and rapeseed containing 80 to 131 members, respectively [32,33,34]. This trend reflects the increasing functional complexity of PP2Cs during plant evolution and the continuous expansion of the biological processes they participate in. Structurally, PP2C family sequences are relatively conserved, with 11 highly conserved catalytic domains. In Arabidopsis, the catalytic domain of most PP2C members resides at the C-terminus, whereas the N-terminus is composed of extension regions of diverse lengths and reduced conservation. It is this sequence diversity that endows PP2Cs with rich regulatory functions. PP2Cs share no amino acid sequence similarity with other protein phosphatases, indicating their relatively independent evolutionary path. Phylogenetic analysis shows that PP2Cs share a common ancestor and are closely related, but during the evolution of plants from prokaryotes to eukaryotes and from unicellular to multicellular organisms, some amino acids or sequences have changed, resulting in diverse structures adapted to different signal transduction requirements [35].

PP2C family members are indispensable in plant stress resistance, actively involved in regulating responses to diverse environmental stresses. The Arabidopsis *AtPP2CG1* gene positively regulates its own salt tolerance and is reliant on ABA [36]. The expression level of the rice *PP2C* gene *OsPP108* significantly increases following salt stress treatment; concurrently, Arabidopsis plants expressing *OsPP108* display markedly improved salt tolerance [37]. The maize *ZmPP2C-A1/A2/A6* genes play a highly significant role in promoting the germination of transgenic Arabidopsis seeds under salinity [38]. The maize *ZmPP2C-A2/A6/A10* genes serve as suppressors in response to water-deficit stress [39]. In contrast, maize plants overexpressing the *ZmPP2C55* gene show significantly improved antioxidant capacity under drought stress, enabling them to better cope with drought conditions and enhance their survival and adaptability [40]. Mutation of the rice *OsPP2C09* gene significantly enhances plant resistance to drought stress, leading to the inference that *OsPP2C09* functions as a suppressor in plant drought tolerance [41]. In contrast, transgenic Arabidopsis overexpressing rice *OsPP108* displays considerably elevated drought tolerance, exhibiting a completely opposite regulatory effect compared to *OsPP2C09* [30]. Apple *MdPP2C24/37* transgenic Arabidopsis plants show reduced drought resistance under stress, negatively regulating drought stress [42]. Collectively, these data imply that under abiotic stress conditions, different PP2C family genes play diverse regulatory roles; some act as positive regulators, while others function as negative regulators. This phenomenon reveals the complexity of their functions and regulatory mechanisms in stressful environments.

This study characterizes the regulatory role of GmPP2C113, a soybean protein phosphatase, in coordinating salt tolerance and symbiotic nodulation. GmPP2C113 was found to be nuclear-localized and function as a transcriptional activator, and interacts with GmPP2C47. Its expression is strongly induced by salt stress and rhizobial inoculation, with highest transcript levels detected in mature nodules. Functional analyses revealed that *GmPP2C113*-overexpressing soybean lines show improved salt tolerance, concomitant with upregulated expression of abiotic stress-responsive genes. Moreover, overexpression of *GmPP2C113* significantly increases nodule number and average fresh weight of nodules per plant under salt stress, along with elevated transcript levels of nodulation marker genes. Collectively, these findings suggest that *GmPP2C113* functions as a positive regulator modulating both salt tolerance and symbiotic nodulation, likely through the activation of stress- and nodulation-related transcriptional programs. This study identifies *GmPP2C113* as a promising candidate gene for improving salt tolerance and symbiotic performance in soybean, providing a potential target for breeding stress-tolerant and nitrogen-efficient soybean varieties with potential applications in sustainable agriculture.

## 2. Materials and Methods

### 2.1. Plant Materials and Growth Conditions

Seeds of cultivated soybean (*G. max* cv. Williams 82) were germinated and grown in moist vermiculite within a light incubator set to 25 °C, and a 16 h light/8 h dark cycle. Roots, stems, and leaves were collected 10 d after sowing, while flowers, seeds, pods, and nodules were collected 42 d after sowing. All samples were snap-frozen in liquid nitrogen and preserved at ultra-low temperature until further analysis, with three biological replicates per treatment.

To analyze the transcriptional characteristics of genes under different abiotic stresses, uniformly grown V1 stage (first trifoliate leaf stage) seedlings were separated into three groups, with 15 plants per group. The three groups of seedlings were, respectively, moved to 1/2 Hoagland solution containing 150 mM NaCl (Solarbio, Beijing, China) for salt stress treatment; transferred to 1/2 Hoagland nutrient solution containing 30% PEG-6000 (Solarbio, Beijing, China) for drought treatment; and the remaining seedlings were cultured in half-strength Hoagland solution and exposed to 4 °C for cold acclimation. For each treatment group, tissue samples were collected at 0 h (pre-stress) and at 3, 6, 12, 24, and 48 h post-treatment, totaling six time points. Root tissues of the seedlings were collected. Samples were snap-frozen in liquid N_2_, stored at −80 °C, and analyzed in triplicate biological repeats.

To identify the effect of rhizobial inoculation on gene expression levels, soybean seeds (W82) were surface-sterilized with chlorine gas and sown in sterile, moist vermiculite. After 5 d, the seedling roots were immersed in a suspension of *Bradyrhizobium diazoefficiens* strain BXYD3 (OD600 = 0.1) for 1–2 h for initial inoculation. Subsequently, seedlings were transplanted into a low-nitrogen nutrient solution for cultivation. Plants were placed in a controlled-environment culture room set to a photoperiod of 13/11 h light/dark (26/24 °C day/night). The nutrient solution was renewed weekly, and its pH was adjusted to 5.8–6.0 with dilute H_2_SO_4_ or KOH as needed. Samples were harvested at scheduled intervals: root tissues at 0 h (before inoculation), 3, 6, 12, 24 h, 3 d, and 7 d post-inoculation; and nodule tissues at 14, 21, 28, 35, 42, and 49 d post-inoculation, totaling 13 samples. All samples were snap-frozen in liquid N_2_ and stored at −80 °C. The experiment was performed with three biological replicates.

To investigate transcriptional responses to combined salinity and rhizobial inoculation, root tissues were harvested 24 h following co-application of 150 mM NaCl and BXYD3 inoculum (15 mL each, final OD600 = 0.1).

*Arabidopsis thaliana* and *Nicotiana benthamiana* plants were cultivated in a controlled culture room at a 24/20 °C (day/night) temperature cycle and a 12 h photoperiod.

### 2.2. Gene Expression Analysis

Primers for qRT-PCR were designed with Primer 5.0 based on the SoyBase sequence *GmPP2C113* (Glyma19G069200; Table S1). RNA was extracted from W82 roots using the MiniBEST Kit (TaKaRa Bio Inc., Otsu, Shiga, Japan) per the manufacturer’s protocol. Gene expression levels were detected following the operating instructions of TaKaRa’s reverse transcription and fluorescence quantitative kits. qRT-PCR was performed on a CFX96 system (Bio-Rad Laboratories, Inc., Hercules, CA, USA). Using *GmACTIN11* as the internal reference, relative gene expression was determined via the 2^−ΔΔCt^ method, with data processed and plotted in Microsoft Excel from three biological and three technical replicates per experiment.

### 2.3. Subcellular Localization of the Protein

The coding sequence of *GmPP2C113* (without the stop codon) was translationally fused to GFP, whereas the nuclear marker NtTGA2.2 (AAF06696) was fused to RFP as a positive control. Arabidopsis protoplasts were co-transformed with GmPP2C113-GFP and NtTGA2.2-RFP, together with a control combination of 35S::GFP and NtTGA2.2-RFP. Following 16 h of dark incubation at 22 °C, fluorescence was observed using a Zeiss LSM880 confocal laser scanning microscope (Carl Zeiss AG, Oberkochen, Baden-Württemberg, Germany).

### 2.4. Transcriptional Activity Assay

The Yeast Gold system (Clontech, Mountain View, CA, USA) was used for the yeast two-hybrid assay. First, the ORF of *GmPP2C113* was amplified and cloned into the BD vector pGBKT7 and the AD vector pGADT7, respectively. Then, the BD-GmPP2C113 and pGADT7 plasmids were co-transformed into the Y2HGold yeast strain, and the AD-GmPP2C113 and pGBKT7 plasmids were co-transformed into the Y2HGold yeast strain. Transformants were plated on DDO and DDO/X/A (with 125 ng mL^−1^ Aureobasidin A and 40 μg mL^−1^ X-α-gal) and incubated at 30 °C for 3–5 d in the dark. BD-53/AD-T and BD-Lam/AD-T were included as positive and negative controls.

### 2.5. In Vivo LCI Assay

*GmPP2C113* and *GmPP2C47* (Glyma09G045100) were cloned into LCI vectors and transformed into *Agrobacterium tumefaciens* GV3101. Single colonies were inoculated into LB liquid medium for amplification culture. After the bacterial culture reached an appropriate concentration, the OD600 value of each suspension was adjusted to 1.0 using infiltration buffer. Subsequently, the two Agrobacterium suspensions carrying different vectors were thoroughly combined in equal volumes. The mixed bacterial suspension was infiltrated into the abaxial epidermis of *N. benthamiana* leaves. Following infiltration, the plants were incubated at 23 °C for 40 h prior to luciferase activity analysis. For LUC detection, the leaves were evenly sprayed with luciferin substrate and kept in darkness for 10 min to minimize background autofluorescence. Chemiluminescence was recorded with an automated imaging system (Tanon-5200, Tanon Science & Technology Co., Ltd., Shanghai, China).

### 2.6. Obtainment of Stable Transgenic Soybean Plants Overexpressing GmPP2C113

The coding sequence of *GmPP2C113* was PCR-amplified and cloned into p1301U (*BamH* I/*Kpn* I) to generate GmPP2C113-OE. Transgenic soybean plants overexpressing *GmPP2C113* were generated via Agrobacterium-mediated cotyledonary-node transformation of cv. *Williams 82*, following the protocol described by Ke et al. [43]. with minor modifications. Briefly, cotyledon explants were inoculated with *A. tumefaciens* strain GV3101 harboring the GmPP2C113-OE construct, co-cultivated for 3 d, and then transferred to shoot induction medium supplemented with glufosinate (10 µM) and antibiotics (timentin, cefotaxime, and carbenicillin) for selection of transgenic events. The glufosinate concentration was increased to 30 µM during subculture and reduced to 15 µM during shoot elongation. Regenerated shoots (3–4 cm) were excised and rooted on medium containing 7.4 µM indole-3-butyric acid (IBA). Rooted T0 plants were transferred to soil matrix and self-pollinated to generate T1 progeny; this selfing process was repeated to obtain T2 and T3 generations.

For selection of transgenic lines, T1 plants were initially screened by painting half of the adaxial leaf surface with 120 mg/L glufosinate, and positive plants were further confirmed by spraying with 1.3 mM glufosinate at the first-trifoliate stage, followed by PCR verification. Homozygosity of the T3 generation was confirmed by segregation analysis of the glufosinate resistance phenotype (progeny showing 100% resistance) and further validated by qRT-PCR to confirm uniform transgene expression across individual plants within each line. Three independent transgenic lines (OE-3, OE-4, and OE-8) exhibiting relatively high *GmPP2C113* transcript levels, as determined by qRT-PCR, were selected for further analyses. Gene expression levels in T3 transgenic plants were detected using primers GmPP2C113-rt (Table S1). Wild-type W82 plants were used as controls in all experiments.

### 2.7. Salt Stress Treatment and Physiological Index Analysis of Transgenic Soybean

T3 generation seeds from the three aforementioned transgenic lines were selected, and seed germination conditions were as described above. After two weeks of normal growth, uniformly growing plants were selected for salt stress experiments: plants were equally allocated into three groups for each transgenic line, and irrigated daily with 1/8 Hoagland nutrient solution containing 0, 150, or 300 mM NaCl. Wild-type soybean plants served as controls. Each treatment group consisted of 20 plants, totaling 12 groups. After one week of salt stress treatment, photographs of the salt tolerance phenotype were taken, and plant height and above-ground dry weight were measured and statistically analyzed.

Leaves from WT and transgenic soybean lines exposed to different salinity levels for one week were collected to determine proline content, malondialdehyde (MDA) content, chlorophyll content, and relative plasma membrane permeability. Additionally, leaf and root tissues from plants under the same treatment conditions were collected to measure Na^+^ and K^+^ contents. The specific determination methods followed those described by Ke et al. [44].

### 2.8. Expression Analysis of Stress-Related Genes

Roots of WT and transgenic soybean lines exposed to 150 mM NaCl for 14 d were harvested for qRT-PCR with the primers shown in Table S1. Relative transcript levels of eight stress-related genes (*GmDHN15*, *GmNHX5*, *GmSOS1*, *GmTGA13*, *GmLEA*, *GmGST1*, *GmWD40* and *GmMYB48*) were evaluated using the 2^−ΔΔCt^ method. Using wild-type plants as controls and *GmACTIN11* as the internal reference, all experiments were carried out with three biological and three technical replicates, and the data were processed and graphed in Microsoft Excel.

### 2.9. Evaluation of Nodulation Phenotype in Transgenic Soybean Under Salt Stress

Wild-type and transgenic soybean seedlings germinated for 7 d in sterile vermiculite were placed in culture pots. Each pot was irrigated with 15 mL of 150 mM NaCl solution and 15 mL of *Bradyrhizobium diazoefficiens* strain BXYD3 suspension (OD600 = 0.1) diluted in low-nitrogen nutrient solution. Subsequently, NaCl treatment and BXYD3 inoculation were repeated every three days. After 14 days of combined salt stress and rhizobial inoculation, nodules were collected from each plant. The number of nodules per plant was counted, and the average fresh weight of nodules per plant was determined immediately after harvest for wild-type (WT) and three independent transgenic lines (OE-3, OE-4, and OE-8) under both normal and salt-stress conditions. Nodules were collected from each line for qRT-PCR quantification of three nodulation markers (*GmENOD40-1*, *Gmcalmodulin* and *GmLb1*) via the 2^−ΔΔCt^ method.

### 2.10. Data Analysis

Microsoft Excel 2010 was used to calculate means and standard errors (SE) for statistical analysis. Data were analyzed using Student’s t-test (Microsoft Excel 2010) for pairwise comparisons or one-way ANOVA (GraphPad Prism 7.0) for multiple-group comparisons.

## 3. Results and Analysis

### 3.1. Molecular Characteristics of GmPP2C113 and Its Interaction with GmPP2C47

**Figure 1:**
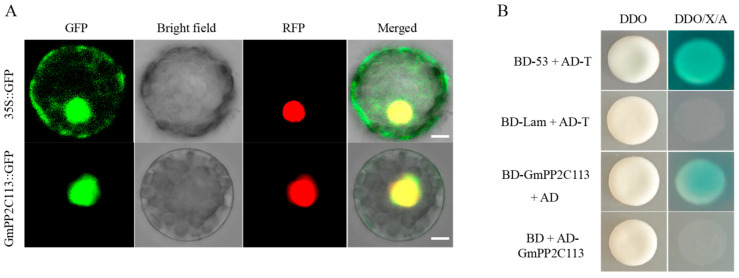
Subcellular localization and transcription activation analysis of GmPP2C113. (**A**) The recombinant plasmids of GmPP2C113-GFP and pCAMBIA1300-35S-GFP were co-transformed with pBI121-mcherry into Arabidopsis protoplast, and the GFP/RFP florescence was observed using a confocal scanning microscope (LSM880). Bars = 25 µm. (**B**) Transcriptional activity analysis of GmPP2C113 in yeast cells. DDO: SD/-Leu/-Trp double dropout medium; DDO/X/A: SD/-Leu/-Trp double dropout medium supplemented with X-α-gal and Aureobasidin A; BD-53/AD-T: positive interaction control; BD-Lam/AD-T: negative control.

For GmPP2C113 localization analysis, a GmPP2C113-GFP fusion construct was generated and co-transformed into Arabidopsis protoplasts along with the nuclear marker NtTGA2.2-RFP as a positive control. The GmPP2C113-GFP fusion protein was exclusively localized to the nucleus, while the GFP control exhibited ubiquitous distribution across the cell (Figure 1A). The nuclear localization of GmPP2C113 aligns with its role as a nuclear protein phosphatase.

Given that GmPP2C113 is a nuclear-localized protein, we employed the yeast two-hybrid system to examine whether it possesses transcriptional activation activity. The results showed that all hybrid combinations grew normally on DDO (SD/-Leu/-Trp) medium. Yeast cells containing the BD-GmPP2C113 vector and the empty AD vector, similar to the positive control, were able to grow normally and formed blue colonies on DDO/X/A medium containing Aureobasidin A and X-α-gal (Figure 1B). This indicates that the GmPP2C113 protein itself possesses transcriptional activation function.

Due to the transcriptional activation activity of GmPP2C113, which precludes the use of the yeast two-hybrid system to identify its interacting target proteins, the firefly luciferase chemiluminescence technique (LCI) was employed to screen for proteins interacting with GmPP2C113 in vivo in *N. benthamiana*. A distinct fluorescence signal was observed in the *N. benthamiana* leaf area co-injected with GmPP2C113-cLUC and GmPP2C47-nLUC, whereas no fluorescence signal was observed in the area co-injected with GmPP2C113-cLUC and GmPP2C72-nLUC (Figure S1). This indicates that GmPP2C113 and GmPP2C47 can physically interact in *N. benthamiana* leaf cells.

Bioinformatics analysis revealed that GmPP2C113 and GmPP2C47 share highly similar sequence regions in their conserved domains, suggesting they may possess similar functions (Figures S2A and S3A). A total of 38 phosphorylation sites were identified in the GmPP2C113 protein, including 29 serine, 8 threonine, and 1 tyrosine residue (Figure S2B). In contrast, the GmPP2C47 protein contains 61 phosphorylation sites, comprising 48 serine, 9 threonine, and 4 tyrosine residues (Figure S3B). The amino acid sequence of GmPP2C113 is 377 residues in length, and its secondary structure primarily consists of alpha helices (35.81%), beta strands (5.57%), and random coils (42.71%) (Figure S2C). The amino acid sequence of GmPP2C47 is 517 residues long, with a secondary structure mainly composed of alpha helices (40.81%), beta strands (2.71%), and random coils (41.78%) (Figure S3C). The secondary structures of both proteins are predominantly composed of alpha helices and random coils, with highly similar distribution proportions of alpha helices, beta turns, and random coils between the two. However, differences exist in the proportions of certain structures, which may reflect their functional divergence.

### 3.2. Expression Patterns of GmPP2C113 in Response to Abiotic Stresses and Rhizobial Inoculation

**Figure 2:**
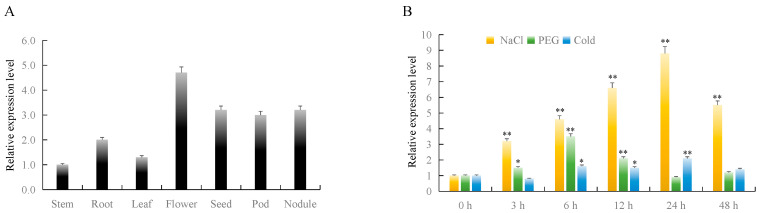
Expression profiles of *GmPP2C113* gene. (**A**) Expression profile of *GmPP2C113* gene in seven different tissues of soybean. (**B**) Expression analysis of *GmPP2C113* gene treated with various abiotic stresses. The expression levels were normalized to that of *GmActin11*. The error bars represent the means ± student error values, and the asterisks indicate a statistical significance (* *p* < 0.05 and ** *p* < 0.01) compared with the corresponding 0 h controls.

Tissue expression pattern analysis showed that the *GmPP2C113* gene was expressed in all examined soybean tissues, with the highest expression level observed in flowers, and relatively high expression levels also maintained in seeds and nodules (Figure 2A). This indicates that *GmPP2C113* exhibits constitutive expression in soybean, but expression levels are tissue-specific. To examine *GmPP2C113* expression in response to abiotic stresses, soybean roots were subjected to salt, drought, and cold treatments. *GmPP2C113* transcript levels were differentially induced by the three stress treatments, with distinct temporal trends observed over time. Notably, under high salt stress, *GmPP2C113* transcript levels increased markedly following treatment, exhibiting an 8.8-fold peak at 24 h compared with the 0 h baseline. In contrast, under drought and low temperature stresses, the gene expression changed more gradually, only showing a slight increase before gradually returning to pre-stress levels (Figure 2B). These findings indicate that *GmPP2C113* may function as a key player in the soybean response to salt stress.

**Figure 3:**
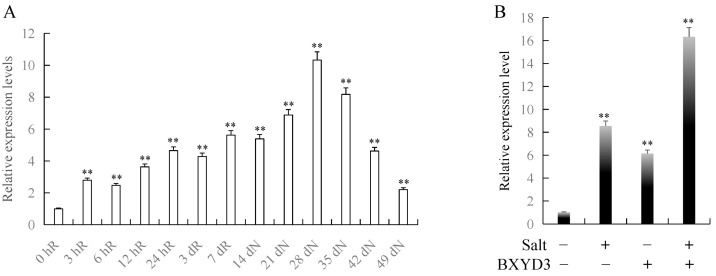
The expression patterns of *GmPP2C113* during soybean nodulation. (**A**) *GmPP2C113* expression in the roots and nodules at different time points after inoculation with BXYD3 at 7 d after soybean sowing. The gene expression levels were normalized to those of GmActin11. All the values are the averages ± SD from three independent experiments. ** *p* < 0.01. (**B**) The expression of *GmPP2C113* was analyzed in soybean roots at 24 h after 150 mM NaCl was combined with the rhizobial BXYD3 inoculation. ** *p* < 0.01.

Root and nodule samples were harvested from soybean at different times after rhizobial inoculation for qRT-PCR analysis of *GmPP2C113* transcript levels. In rhizobia-inoculated roots, *GmPP2C113* expression was significantly upregulated, whereas in nodules, its transcript levels exhibited a temporal pattern of an initial increase followed by a decline, peaking at 28 days post-inoculation before returning to levels comparable to those at the early stage of inoculation (Figure 3A). These findings suggest that the *GmPP2C113* gene is induced by rhizobia and maintains a relatively high expression level during nodule maturation, suggesting its potential involvement in the modulation of nodule development.

Furthermore, the effect of rhizobial inoculation under salt stress conditions on *GmPP2C113* gene expression was analyzed. The results showed that under combined treatment with 150 mM NaCl and rhizobia, the expression level of *GmPP2C113* was significantly elevated, and it was higher than that under salt treatment alone or rhizobial inoculation alone (Figure 3B). These findings imply that *GmPP2C113* may play a role in the regulation of symbiotic nodulation under saline conditions.

### 3.3. Overexpression of GmPP2C113 Enhances Salt Tolerance in Transgenic Soybean

**Figure 4:**
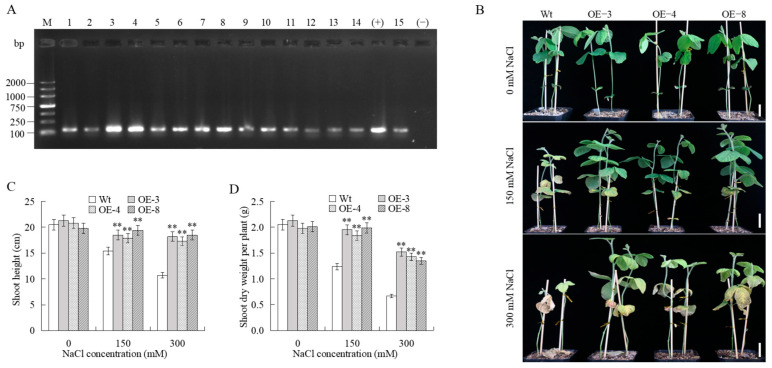
Salt stress phenotype analysis of transgenic soybean overexpressing *GmPP2C113*. (**A**) Semi-qRT-PCR analysis of *GmPP2C113* expression levels in T3 transgenic soybean lines. (**B**) Response of the wild type and three transgenic soybean lines to saline conditions. (**C**) Shoot height was measured 14 d after salt treatment. (**D**) Shoot dry weight per plant was measured 14 d after salt treatment. M: DL2000 Plus DNA Marker; 1-15: Transgenic lines; (+): Plasmid positive control; (−): Plasmid negative control; Wt: Wild type control; OE-3, OE-4, and OE-8: transgenic soybean lines 3, 4, and 8, respectively. Bars = 25 mm. All values are presented as means of three independent replicates (*n* = 30). The error bars indicate SD. *p*-values were calculated using Student’s *t*-test. ** *p* < 0.01.

Given the strong induction of *GmPP2C113* expression by salt stress and rhizobial inoculation, we next investigated its biological function by generating transgenic soybean lines overexpressing *GmPP2C113*. Overexpressing *GmPP2C113* transgenic soybean lines were successfully obtained using the *A. tumefaciens* GV3101-mediated soybean cotyledonary node stable transformation method. qRT-PCR was performed to examine *GmPP2C113* transcript levels in the roots of different T3 transgenic lines. Three lines (OE-3, OE-4, and OE-8) with elevated transcript levels were chosen for further study (Figure 4A).

Three overexpression lines and WT plants were cultured under normal conditions for 2 weeks, followed by 1 week of treatment with 0, 150, or 300 mM NaCl. Under normal conditions, all lines grew well; however, under 150 mM NaCl stress, the OE lines remained healthy, whereas WT plants exhibited chlorosis and wilting in their leaves. Under 300 mM NaCl stress, the overexpression lines only showed yellowing and drying of basal leaves, with apical leaves remaining normal; in contrast, wild-type plants displayed severe leaf drying and abscission, and plant growth was significantly inhibited (Figure 4B). Across all salt concentrations, the overexpression lines exhibited significantly greater plant height and above-ground dry weight than the wild-type control (Figure 4C,D). Collectively, these results show that *GmPP2C113* overexpression markedly enhances salinity tolerance in soybean.

### 3.4. Overexpression of GmPP2C113 Alleviates Salt-Induced Physiological Damage

**Figure 5:**
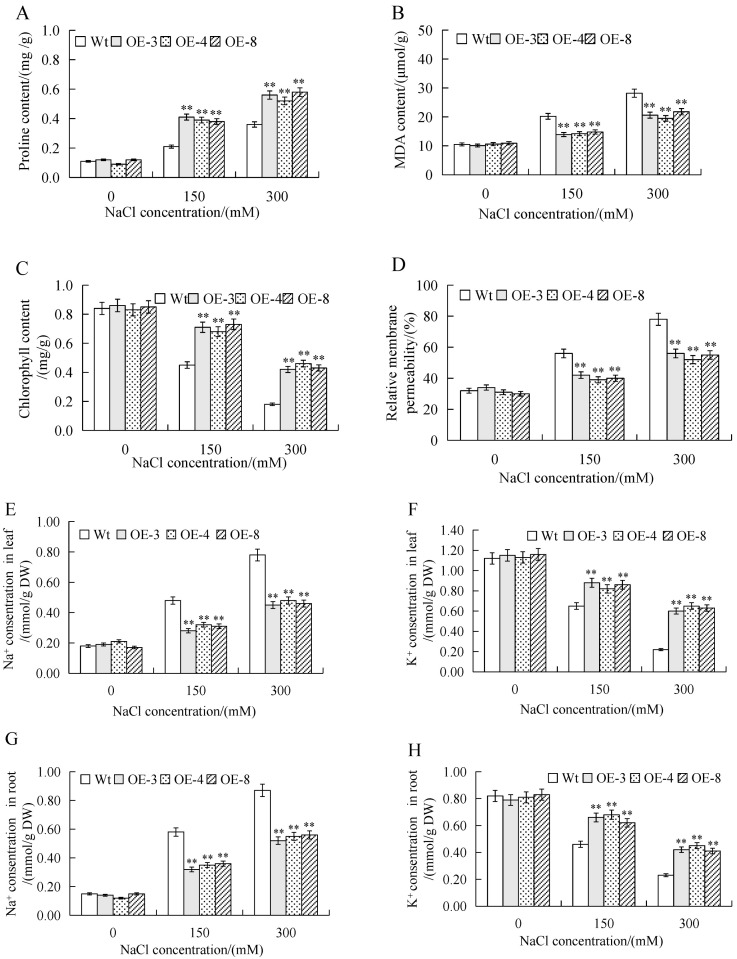
Analysis on physiological indexes of transgenic soybean overexpressing *GmPP2C113* under salt stress. (**A**) Proline content of the wild type and transgenic soybean lines were measured 7 d after treatment. (**B**) MDA content; (**C**) Chlorophyll content; (**D**) Relative membrane permeability; (**E**) Na⁺ concentration in leaf; (**F**) K⁺ concentration in leaf; (**G**) Na⁺ concentration in root; (**H**) K⁺ concentration in root. *n* = 15. All values are presented as means of three independent replicates. The error bars indicate SD. *p*-values were calculated using Student’s *t*-test. ** *p* < 0.01 compared with Wt.

To further characterize the physiological response of transgenic soybean to salt stress, proline content was measured in leaves. In the absence of stress, transgenic and wild-type plants exhibited comparable performance. After salt stress treatment, proline content in both increased with increasing salt concentration, but the accumulation in OE lines was substantially greater than in WT (Figure 5A), indicating that overexpression of the *GmPP2C113* gene promotes proline accumulation under saline conditions, contributing to the sustaining of cellular osmotic balance. Chlorophyll content analysis showed that with increasing salt concentration, chlorophyll content in both transgenic lines and wild-type exhibited a downward trend. However, transgenic lines retained substantially greater chlorophyll content than the wild-type (*p* < 0.01), with a notably smaller decrease relative to the control (Figure 5C), suggesting that overexpression of *GmPP2C113* helps maintain photosynthetic capacity under stress.

Analysis of cell membrane damage-related indicators revealed that in the absence of stress, leaf malondialdehyde (MDA) content and relative plasma membrane permeability were comparable between OE and WT plants. After salt stress, both MDA content and membrane permeability increased in both, but the increase in transgenic lines was significantly lower than that in the wild-type (*p* < 0.01) (Figure 5B,D), indicating that overexpression of *GmPP2C113* can mitigate oxidative damage to cell membranes caused by salt stress and enhance membrane stability.

Further determination of Na^+^ and K^+^ contents showed that under control conditions, Na^+^ and K^+^ levels in leaves and roots did not differ significantly between OE lines and WT. As NaCl concentration increased, Na^+^ content in both increased, but the increase in transgenic plants was markedly lower than that in the control. Simultaneously, K^+^ content in both decreased, while the decrease in transgenic plants was smaller (Figure 5E–H). This indicates that overexpression of *GmPP2C113* can enhance the plant’s ion homeostasis regulation capacity by reducing Na^+^ accumulation and maintaining higher K^+^ levels.

In summary, overexpression of *GmPP2C113* substantially enhances salt resistance in soybean by promoting proline accumulation, maintaining photosynthetic capacity, alleviating oxidative damage, and regulating ion balance.

### 3.5. Overexpression of GmPP2C113 Upregulates Stress-Related Genes Under Salt Stress

**Figure 6:**
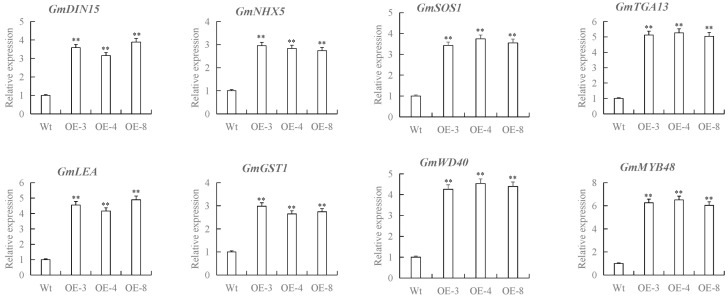
Expression of stress-related genes as assessed by qRT-PCR under salt stress. Data are expressed as the mean ± SD of three replicates. Asterisks indicate a significant difference between Wt and transgenic plants. ** *p* < 0.01.

To explore the molecular role by which GmPP2C113 participates in the salt stress response, eight abiotic stress-related genes: *GmDHN15*, *GmNHX5*, *GmSOS1*, *GmTGA13*, *GmLEA*, *GmGST1*, *GmWD40*, and *GmMYB48* were selected to analyze their expression characteristics in the roots of transgenic soybean. qRT-PCR confirmed that these eight stress-responsive genes were significantly induced in transgenic roots compared with WT under salt stress (Figure 6). This indicates that overexpression of *GmPP2C113* can induce the upregulation of multiple downstream stress-responsive genes, suggesting that *GmPP2C113* likely contributes to salt tolerance in transgenic soybean by upregulating these stress-related genes.

### 3.6. Overexpression of GmPP2C113 Promotes Symbiotic Nodulation Under Salt Stress

**Figure 7:**
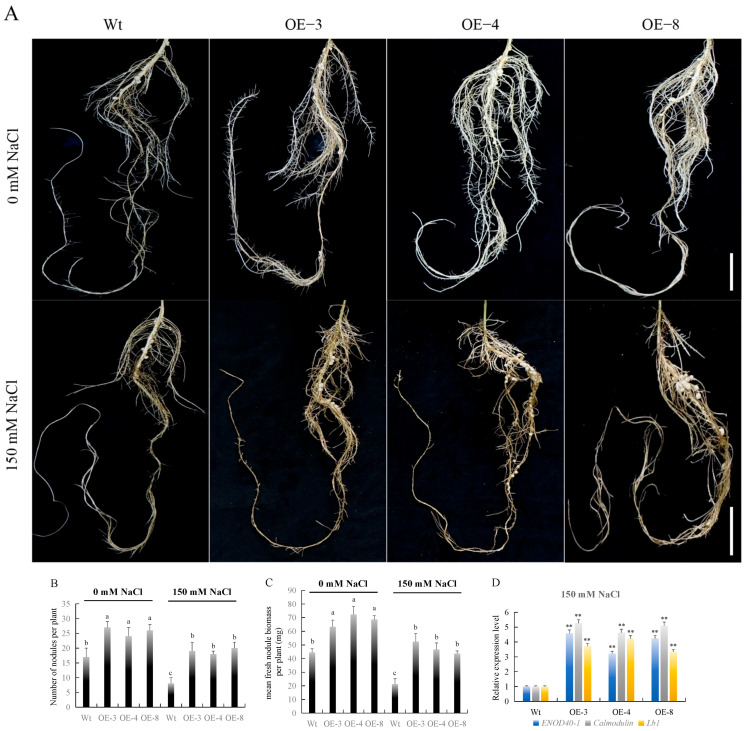
*GmPP2C113* promotes soybean nodule formation under salt stress. (**A**) Nodulation phenotype of transgenic soybean roots with wild type (Wt) at 14 d after inoculation (DAI) under normal condition or salt stress; Bar, 1 cm. (**B**) Nodule number of transgenic soybean roots at 14 days after inoculation (DAI) under conditions without or with 150 mM NaCl (*n* = 15). Different lowercase letters indicate extremely significant differences among different treatment times (*p* < 0.01). (**C**) Average fresh weight of nodules per plant (mg) of transgenic soybean roots at 14 DAI under conditions without or with 150 mM NaCl (*n* = 15). Different lowercase letters indicate extremely significant differences among different treatment groups. (**D**) The transcript level of *ENOD40-1*, *Calmodulin* and *Lb1* was analyzed in transgenic roots and Wt under salt stress (150 mM NaCl) at 14 DAI in soybean (** *p* < 0.01).

To investigate the effect of salinity on the nodulation capacity of transgenic soybean overexpressing *GmPP2C113*, this study analyzed the nodulation status of transgenic and wild-type plants at 14 d post-rhizobial inoculation under 0 and 150 mM NaCl conditions. Transgenic lines had markedly more nodules than WT under control conditions (*p* < 0.01). Although salt stress reduced nodulation in both lines, transgenic nodules remained substantially more abundant than those of WT (*p* < 0.01) (Figure 7A,B). The average fresh nodule weight per plant was significantly higher in the OE lines compared with WT under both normal and salt-stress conditions (Figure 7C). These findings suggest that *GmPP2C113* overexpression sustains nodulation capacity in soybean under salinity.

Further detection of the transcript abundance of nodulation-related genes showed that under salt stress conditions, the transcript levels of *GmENOD40-1*, *GmCalmodulin*, and *GmLb1* in the mixed root and nodule samples of transgenic soybean were significantly upregulated (Figure 7D). This indicates that overexpression of *GmPP2C113* can stimulate the expression of nodulation marker genes, thereby promoting the symbiotic nodulation process in soybean.

## 4. Discussion

Soil salinization is a major limiting factor for soybean production. Under saline–alkali stress, plants activate adaptive responses via ABA signaling, in which PP2Cs act as core negative regulators by interacting with PYR/PYL/RCAR and SnRK2 [31]. Recent studies have revealed that PP2Cs can also form interaction networks with other family members, thereby exerting more nuanced regulatory effects [42]. However, whether and how PP2Cs coordinate salt tolerance and symbiotic nodulation in soybean remains largely unknown.

Herein, a PP2C family member, *GmPP2C113*, was cloned from soybean, and its molecular characteristics and biological functions were systematically analyzed. GmPP2C113 was found to localize to the nucleus and exhibit transcriptional activation activity (Figure 1). This provides direct molecular evidence for its involvement in salt stress signal transduction. To further elucidate its regulatory network, a tobacco split-luciferase complementation (LCI) assay was employed to detect the interaction between GmPP2C113 and GmPP2C47. The LCI assay revealed a physical association between GmPP2C113 and GmPP2C47 in *N. benthamiana* leaf cells (Figure S1). However, we acknowledge that further validation using complementary approaches, such as co-immunoprecipitation (Co-IP) in transgenic soybean lines, is required to confirm the biological relevance of this interaction under native conditions. Notably, these two proteins are homologous and exhibit high conservation in key functional domains (Figures S2 and S3), suggesting that they may regulate protein phosphorylation levels through similar biochemical mechanisms. We therefore hypothesize that GmPP2C113 and GmPP2C47 may coordinately participate in the soybean response to saline–alkali stress. Collectively, this study provides evidence for the formation of protein complexes between different PP2C family members in soybean, offering new insights into the functional diversity and interaction networks of PP2Cs.

PP2C protein phosphatases, through their interaction networks with kinases, transcription factors, and other signaling molecules, finely regulate plant responses to internal and external environmental signals, thereby influencing growth and developmental processes. Current research has confirmed that the PP2C family is widely involved in important signaling pathways, including ABA-mediated signaling, the MAPK cascades, the salt overly sensitive (SOS1) pathway, and CBL-interacting protein kinase (CIPK) pathways [45,46,47]. Future research urgently needs to clarify whether PP2Cs can act as key molecular switches, coordinately activating multiple signaling pathways to achieve precise regulation of plant stress responses. Furthermore, as important members of the phosphatase family, PP2Cs primarily regulate the activity of downstream proteins through dephosphorylation modification. How these two mechanisms work together synergistically, and how the regulatory mechanisms of PP2C’s own activity (such as post-translational modifications, protein interactions, etc.) are achieved, remain to be elucidated. In-depth analysis of these scientific questions will provide key theoretical foundations for unraveling the molecular mechanisms of plant stress adaptation.

Transcriptional profiling revealed that *GmPP2C113* was significantly induced by salt stress, reaching its peak expression in roots at 24 h post-treatment (Figure 2), indicating that it may mediate early salt stress responses. Overexpression of *GmPP2C113* significantly enhanced the salt tolerance of transgenic soybean, with plant height and above-ground dry weight significantly higher than WT under high-salinity conditions (Figure 4). *GmMYB48* is a MYB transcription factor that activates stress-responsive genes and confers stress resistance [48]. GmWD40 is a WD40 scaffold protein that mediates protein–protein interactions, thus assisting in normal protein function and participating in abiotic stress responses [49]. GmDHN15 is a drought-responsive dehydrin protein [50]. GmLEA is a late embryogenesis-related gene that enhances abiotic stress tolerance [51]. GmGST1, a GST family member, promotes stress resistance via redox homeostasis maintenance [52]. The significant upregulation of these stress-responsive genes in transgenic roots (Figure 6) further supports the role of *GmPP2C113* in enhancing salt tolerance through regulation of downstream genes.

The efficiency of soybean symbiotic nitrogen fixation is collectively determined by rhizobial infection, nodule organogenesis, and mature nodule fixation activity. However, the entire nodulation process is susceptible to salt stress. Therefore, elucidating the molecular basis of soybean-rhizobia symbiosis and its salt stress response is of great significance for the enhancement of soybean symbiotic efficiency. Previous studies have shown that *GmNAC181* is a critical regulatory factor maintaining the nodulation process under salt stress, enhancing the tolerance of symbiotic nodulation under salt stress by upregulating *GmNINa* and its downstream genes [22]. Unlike previous studies, this research revealed that *GmPP2C113* maintains a relatively high expression level in nodules and is induced by rhizobia (Figure 3A). Inoculation with rhizobia under salt stress further increased the expression of *GmPP2C113* (Figure 3B). Transgenic lines overexpressing this gene maintained a higher number of nodules and greater average fresh weight of nodules per plant under salt stress conditions, along with upregulated expression of nodulation marker genes *GmENOD40-1*, *GmCalmodulin*, and *GmLb1* (Figure 7). Our findings identify *GmPP2C113* as a positive regulator that integrates salt tolerance and symbiotic nodulation at the transcriptional level, and suggest its potential role in sustaining nodulation in soybean under salt stress. Future studies employing gene knockout or knockdown approaches will be instrumental in further validating the biological functions of *GmPP2C113* and *GmPP2C47* in the soybean response to saline–alkali stress. In addition, transcriptomic and proteomic analyses may help to further elucidate their regulatory roles in ABA signaling and symbiotic nodulation, though we acknowledge that loss-of-function analyses will be required to definitively establish the essentiality of *GmPP2C113* in these processes.

In summary, our findings uncover a novel role for *GmPP2C113* in coordinating nodulation and stress response signaling under salt stress. *GmPP2C113* not only stimulates nodulation in the absence of stress but also sustains root development and symbiotic performance under salinity. This discovery provides a potential breeding approach for improving soybean nitrogen fixation and yield via *GmPP2C113* under salt stress. Given the conserved nature of PP2C in legumes, our research offers a conceptual framework for genetic improvement of symbiotic nitrogen fixation in other legume crops.

## 5. Conclusions

In summary, this study identifies *GmPP2C113* as a positive regulator of both salt tolerance and symbiotic nodulation in soybean. *GmPP2C113* is induced by salt stress and rhizobial infection, and its overexpression enhances salt tolerance while promoting nodulation under salt stress. Although GmPP2C113 showed physical interaction with GmPP2C47 in LCI assays, the functional significance of this interaction remains to be determined. These findings suggest that *GmPP2C113* plays a regulatory role in coordinating stress adaptation and symbiosis, and represents a candidate gene for improving soybean performance under saline conditions.

## Data Availability

The original contributions presented in this study are included in the article/Supplementary Material. Further inquiries can be directed to the corresponding author.

## Supplementary Materials

The following supporting information can be downloaded at https://www.mdpi.com/article/10.3390/genes17070815/s1, Table S1. Primers used in this study. Figure S1. Detection of the interaction between GmPP2C113 and GmPP2C47 proteins in tobacco leaves by LCI. Figure S2. Analysis of the conserved domain (A), phosphorylation sites (B) and secondary structure prediction (C) of GmPP2C113. Figure S3. Analysis of the conserved domain (A), phosphorylation sites (B) and secondary structure prediction (C) of GmPP2C47.
