# Supplementary material for: GmPP2C113, a Soybean Protein Phosphatase, Positively Regulates Both Salt Tolerance and Symbiotic Nodulation
Source: Genes (Basel). 2026 Jul 17;17(7):815. doi: 10.3390/genes17070815 (PMC13410229; doi:10.3390/genes17070815)
Supplement: Supplementary file 1 [file genes-17-00815-s001.zip › genes-4399987-supplementary.pdf]

Table S1. Primers used in this study.

Primer name	Forward primer (5'-3')	Reverse primer (5'-3')
GmPP2C113-GFP	CGGGGTACCATGAAGACGCCAAAACG	TGCTCTAGATCAATCAGTTGTTACTG
GmPP2C113-BD	CATGCCATGGATGAAGACGCCAAAACG	GCGTCGACTCAATCAGTTGTTACTG
GmPP2C113-AD	CCCATATGATGAAGACGCCAAAACG	CGGGATCCTCAATCAGTTGTTACTG
GmPP2C113-cLUC	GGGGTACCATGAAGACGCCAAAACG	GCGTCGACTCAATCAGTTGTTACTG
GmPP2C47-nLUC	GGGGTACCATGCTTGAATATGAC	CGGGATCCCTAGAGGCAAATTG
GmPP2C72-nLUC	GGGGTACCATGGCTGGAATTTGC	CGGGATCCTTAATTGGAGTTTGATG
GmPP2C113-OE	GGGGTACCATGAAGACGCCAAAACG	CGGGATCCTCAATCAGTTGTTACTG
GmPP2C113-rt	ACCAGAAGTGACAGTGACCC	GATTCCCAACTCCATCGCAC
GmActin11-rt	GAGCTATGAATTGCCTGATGG	CGTTTCATGAATTCCAGTAGC
GmDIN15-rt	TTTTGTTTTGTTGTATTGTGTAG	GAAAAATCCTCCACCTGACGA
GmNHX5-rt	GTCTGGGTTCAGTCTCGCAC	ATCAGAAAGAGCAAGCCACCA
GmSOS1-rt	TTGTGCTGCATTTCTTCGAG	CGTGCTTCTTCTCCTTCCAC
GmTGA13-rt	GGAGCAACAGTTAGTGGGTATCAC	CTTCCATGCCCTGAGACAAAGC
GmLEA-rt	GGTGGGTGAAACCGCACAAGA	ATGGATGCCGCCACTCCGCCAG
GmGST1-rt	CACAATGAGCAGCCCATAGCA	CTTCAACATTCTTCTCACGCTCT
GmWD40-rt	TGCCAGTCTCGTTAGGCTTTTC	CTTATTGAGTTGTTGTTTGGCAG
GmMYB48-rt	AACAACACTCTTCAGCCAGTTT	GGGCAAAACAAACTTTCCTCAT
GmENOD40-1-rt	CACTTCCTGATACCCGTGAAA	CGCCACTCAAGAAAGAATGTT
GmCalmodulin-rt	TCTCCCAGTCCAAGATCACC	GCCGATATTTTCCCATCTCC
GmLb1-rt	CTCCAAGCCCATGCTGAAAA	TGGCATCTGCAAGTGTCACTTC

The underline region indicates the restriction enzyme.

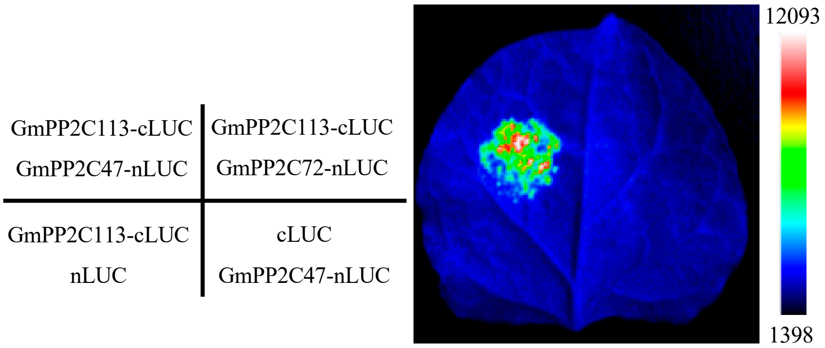

Supplementary Figure S1. Detection of the interaction between GmPP2C113 and GmPP2C47 proteins in tobacco leaves by LCI.

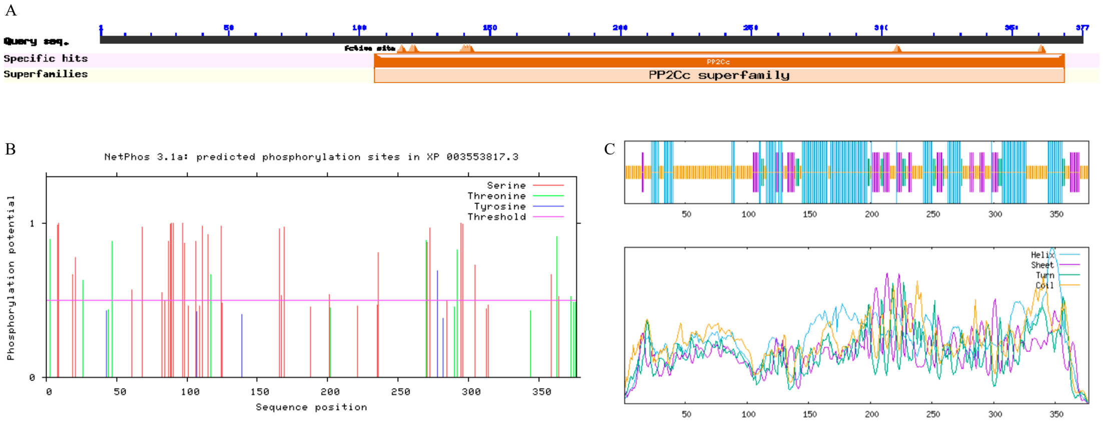

Supplementary Figure S2. Analysis of the conserved domain (A), phosphorylation sites (B) and secondary structure prediction (C) of GmPP2C113.

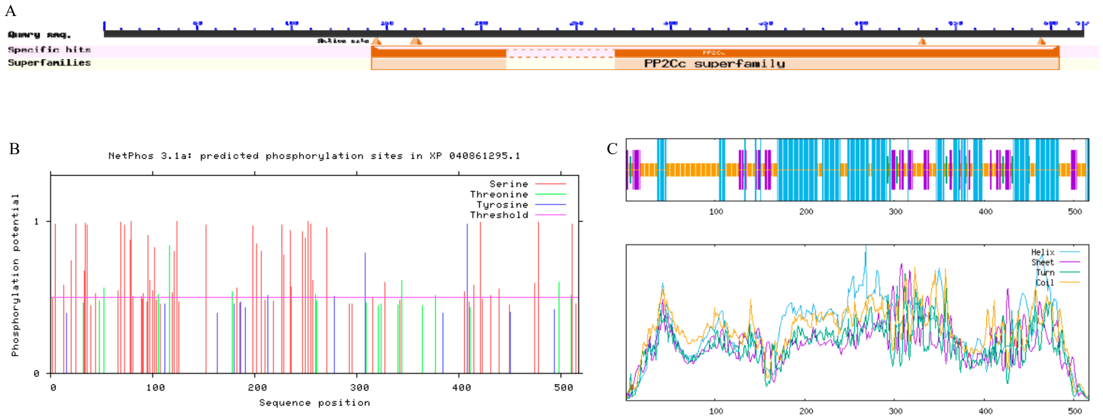

Supplementary Figure S3. Analysis of the conserved domain (A), phosphorylation sites (B) and secondary structure prediction (C) of GmPP2C47.
